# Strategies for Aspiring Biomedical Researchers in Resource-Limited Environments

**DOI:** 10.1371/journal.pntd.0000274

**Published:** 2008-08-27

**Authors:** Patricia J. Garcia, Walter H. Curioso

**Affiliations:** 1 School of Public Health and Administration, Universidad Peruana Cayetano Heredia, Lima, Peru; 2 School of Medicine, Universidad Peruana Cayetano Heredia, Lima, Peru; London School of Hygiene & Tropical Medicine, United Kingdom

## Introduction

Countries struggling with global health challenges desperately need local biomedical researchers to find health care solutions to address the deadly diseases that affect their populations. Building the scientific capacity of resource-limited countries is a clear priority among the scientific community [1]–[3].

As the Global Forum for Health Research Report stated [4], “Strengthening research capacity in developing countries is one of the most effective and sustainable ways of advancing health and development in these countries and of helping correct the 10/90 gap in health research.” The 10/90 gap refers to the statistical finding of the Global Forum for Health Research that only 10% of all global health research funding is directed to research on the health problems that affect 90% of the world's population [5].

Great efforts are now being made to correct this gap, and some call it the golden age of global health. Many researchers in resource-limited environments have the opportunity to train outside their country and are offered scholarships to do so, with the goal that they will return and help their country. For researchers willing to deal with developing world challenges (poor infrastructure and support), there are exciting opportunities to solve a nation's most pressing health problems and make a name for themselves along the way.

In this paper, we will share some key strategies for aspiring biomedical researchers based on the experiences of researchers affiliated with Universidad Peruana Cayetano Heredia (UPCH) in Lima, Peru—a major hub for global health training in South America.

## Discussion: Strategies for Aspiring Biomedical Researchers

Faced with a lack of research funding, poor research facilities, limited career opportunities, threats of violence, and a culture not supportive of evidence-based decision-making, it's easy to see why fleeing the country and joining the brain drain is a real possibility [6],[7]. But even in countries with the most limited resources, high-quality and meaningful research can be done [8], and the results may not be highly appreciated but can be highly visible.

Conducting research in such settings takes lots of patience. Approvals are cumbersome and time-consuming, and additional, unexpected approvals might be required later in the process. And there are often high-level changes and turnover in governmental offices, which can directly affect research projects. While science is increasingly performed by research groups rather than by individual scientists [9], and researchers often benefit from working as part of a multidisciplinary team [10], working in teams is not easy. Differences can cause team members to contest or challenge one another's contributions, although these differences also enrich collaboration [10]. Cooley suggests that interdisciplinary teams can experience problems with group interaction because of a lack of organizational procedures, miscommunication, misunderstanding, and inadequate commitment [11], so it's important to take action to keep the project moving forward

## Strategies to Apply

### Partnerships/Collaborations

For new biomedical researchers, the best way to find collaborators is by asking those in your country who have conducted research or who have published with other colleagues. Professors at medical schools are often principal investigators in clinical trials and will recruit doctors to work with them for little or no money. Find out what outside universities these professors are linked with to see how connected they are to outside collaborators. There are also networks of researchers that you can consult for advice. For example, at UPCH we developed a Global Health Task Force made up of professors and researchers from different schools (medicine, public health, sciences, education, dentistry, and veterinarian medicine) interested in the advancement of global health in Peru [12]. This group meets periodically to discuss curriculum issues on global health to be taught at UPCH. Collaborating with policy makers is also vital because it ensures relevance of research priorities and application of findings.

### Focus

Look for a topic you would like to spend most of your life working on so you will be passionate about the study. Each project is like a brick for building bigger projects in this area, and results from an initial study will help you get funding for other studies. Studies that address the major burdens of disease in your own country, such as malnutrition, tuberculosis, and HIV/AIDS, may be given funding preference, but consider other areas that may be important in global health.

### Innovation

With limited resources, you have to know how to do more with less, but this allows you to be innovative. For example, when a Bolivian researcher wanted to create a microcentrifuge, he used a blender, aluminum bowl, and water-tap adapters [5]. In Peru, scientist Leon-Barua developed a system to diagnose meteorism (a distention of the abdomen resulting from the accumulation of gas or air in the intestine or peritoneal cavity) in people by using a simple system of three vases to study feces [13],[14].

### Searching for Funding

Funding is by far a researcher's biggest challenge, but there are resources. Box 1 shows selected funding agencies in international health. The Community of Science (http://fundingopps.cos.com/) has a searchable database representing more than 400,000 funding opportunities worth more than US$33 billion. At the midway point through a current grant, or even before, always keep looking for more funding opportunities. Many times funds are available (specially at national agencies) but go unused because there is a lack of good proposals. Don't get discouraged; researchers all over the world have problems getting funding.

### Box 1. List of Selected Funding Agencies in International Health

#### International Agencies

- Fogarty International Center/US National Institutes of Health (http://www.fic.nih.gov/)
- The Bill & Melinda Gates Foundation (http://www.gatesfoundation.org/)
- The Canadian International Development Agency (http://www.acdi-cida.gc.ca/)
- The Department for International Development (http://www.dfid.gov.uk/)
- The Global Fund (http://www.theglobalfund.org/)
- The International Development Research Centre (http://www.idrc.ca/)
- The Pan American Health Organization (http://www.paho.org/)
- The Rockefeller Foundation (http://www.rockfound.org/)
- The Wellcome Trust (http://www.wellcome.ac.uk/)
- The World Bank (http://www.worldbank.org/)
- United States Agency for International Development (http://www.usaid.gov/)
- World Health Organization's Special Programme for Research and Training in Tropical Diseases (http://www.who.int/tdr/)

#### National Agencies

- National Institute of Health of your own country (e.g., the Peruvian National Institute of Health, available at: http://www.ins.gob.pe/)
- National Councils of Science and Technology of your own country (e.g., the Peruvian National Council of Science and Technology, available at: http://www.concytec.gob.pe/)
- Some nongovernmental organizations fund proposals, depending on the topic of interest

### Sharing

Share your work in every possible way. Present at national and international conferences and show how to apply your research findings in-country. According to a survey of physicians in secondary and tertiary hospitals and developing countries, health care institutions are more likely to change practices if the research is locally based [15]. Know how to talk about your project in an understandable fashion that inspires your audience, especially authorities, policy makers, and funding agencies. Academic professionals in developing countries tend to work in relative isolation from primary care settings, and fewer still interact with public health policy makers [16], so make an effort to share with these crucial audiences.

### Publishing

Publishing is one of the best ways to share your results with the international community and the way your scientific production will be measured. Usually you do not receive funds for publishing, but it does help your chances of getting future funding. If you don't publish, the likelihood of getting funded in the future is low. The most important thing is to get your ideas on paper, in whatever form, as a starting point. It doesn't have to be pretty or in good English—just get the concepts down to begin with and be patient with the editing process. You can publish in your own language, but it is essential to submit to an international journal as well. Before you start writing, decide the order of authors and how each person will contribute. Ask for help from others and don't get discouraged. People are more than happy to edit.

### Teaching

Teaching is important, but do not let it overwhelm you. If you are in an academic environment, try to negotiate the time you need to conduct your research and point out the overhead or administrative funding that the researcher brings to the university when he/she gets a grant. Don't let teaching take over too much of your research time and duties, and include your research in your teaching. Consider mentoring another researcher; if you are a new faculty member, look for a mentor for yourself. One of the best ways to find a mentor is by asking those inside or outside your university who have conducted research or published on the same topic as you.

### Promotion

Promote an open discussion of research in your institution, including the role of research in improving public health in the community. Promote capacity development through informatics grants, training grants, and other grants that aim to build up a laboratory or library. Promote and organize seminars disseminating your research so you can attract potential collaborators. Create a Web site to promote your research team, including an English version.

## Issues That Might Arise

### Ethical Issues

In many resource-limited environments, ethical issues related to human research participants might be difficult and time-consuming, but the protection of human participants is an important topic and better practices are needed in research work [17]. In addition, many places lack institutional review boards, and there are not many trained personnel to deal with these issues. The Fogarty Bioethics Program (http://www.fic.nih.gov/programs/training_grants/bioethics/index.htm) is one of the few programs where you can get training in ethics. Try to organize a course, symposium, or conference to address ethical issues.

### Administrative Support

Human resources and infrastructure for the administrative, governance, financial, and management functions are key for institutions to deliver research excellence. You might find administrative support within your research facility. If not, try to create your own administrative system as an example to encourage your institution to create an official system. For example, the research and development project office at UPCH (a division of the Vice-Rectorate of Research) was created in 2002 and provides administrative support to researchers at the university (http://www.upch.edu.pe/vrinve/).

### Gender Issues, Family, and Research

Women often face special challenges as aspiring researchers, due not only to cultural issues but also to additional responsibilities like motherhood. Women might get together and form informal support groups for each other, or tenured women faculty might “adopt” junior women and guide them through tenure, helping to keep them focused. Create a support network.

### Incentives to Return

Finally, institutions could apply some strategies to encourage researchers to return after training and stay in a developing setting. For example, institutions affiliated with the AIDS International Training and Research Program ask trainees to sign a “condition of appointment” or a return agreement prior to training [6]. This can be a valuable tool to encourage trainees to return home. However, this strategy cannot guarantee a researcher will return. One idea to encourage researchers to return is to promote re-entry grants, which are funds to help researchers to re-establish themselves and find their niches after some time in other countries.

Some institutions, like UPCH, institutionalized a grant for Ph.D.s returning to Peru to work at the university, which pays their salary for one to two years at a relatively high level compared to other university positions. In exchange, these returning Ph.D.s commit to working in research and looking for funding opportunities. We have found this experience very successful in transitioning these valuable researchers back to their home country.

## Conclusion

While starting out in research is not easy, it's always rewarding. Ask yourself: how do you see yourself in five to ten years, professionally? What would you need to do to reach that goal? Part of the process is to develop a critical mass of peer researchers that could push forward all the different activities to promote research in your environment (i.e., training, ethics, administration, advocacy, communication, fundraising, etc). Define some benchmarks. Remember that while this work can be a struggle and time-consuming, success is only that much sweeter. And any progress, no matter how small, is a step forward in building the health of a nation.

## Supporting Information

Alternative Language Abstract S1 Spanish Translation of the Abstract by Walter H. Curioso
(0.03 MB DOC)
