# Supplementary material for: Strategies for Aspiring Biomedical Researchers in Resource-Limited Environments
Source: PLoS Negl Trop Dis. 2008 Aug 27;2(8):e274. doi: 10.1371/journal.pntd.0000274 (PMC2565775; doi:10.1371/journal.pntd.0000274)
Supplement: Alternative Language Abstract S1 — Spanish Translation of the Abstract by Walter H. Curioso (0.03 MB DOC) [file journal.pntd.0000274.s001.doc]

**Estrategias para Nuevos Investigadores Biomédicos en Entornos con Recursos Limitados**

Patricia J. Garcia ^1^*, Walter H. Curioso ^1,2^

1. Facultad de Salud Pública y Administración. Universidad Peruana Cayetano Heredia, Lima, Perú.

2. Facultad de Medicina. Universidad Peruana Cayetano Heredia, Lima, Perú.

* Correspondencia. E-mail: pattyg@u.washington.edu

**Resumen**

Fortalecer la capacidad de realizar investigación en los países en vías de desarrollo es uno de los caminos más efectivos y sostenibles en el avance y desarrollo de la salud de éstos países, además de ayudar a corregir el destino de las inversiones en la mayoría de temas de salud mundial. Los investigadores biomédicos, deseosos de enfrentar los desafíos del mundo en desarrollo, tienen oportunidades interesantes al frente de la salud global para realizarse y hacerse de un nombre. En este trabajo compartimos algunas estrategias claves para los nuevos investigadores biomédicos en países de bajos y medianos recursos.
